# Single-stage surgical modification for neonatal aortic coarctation and ventricular septal defect: a case report

**DOI:** 10.1093/ehjcr/ytag098

**Published:** 2026-02-25

**Authors:** Xinmeng Yang, Pengchao Xing, Kefeng Hou, Rui Chen, Silin Pan

**Affiliations:** Heart Center, Qingdao Women and Children's Hospital, Qingdao University, No. 6 Tongfu Road, Shibei District, Qingdao, Shandong Province 266000, China; Heart Center, Qingdao Women and Children's Hospital, Qingdao University, No. 6 Tongfu Road, Shibei District, Qingdao, Shandong Province 266000, China; Heart Center, Qingdao Women and Children's Hospital, Qingdao University, No. 6 Tongfu Road, Shibei District, Qingdao, Shandong Province 266000, China; Heart Center, Qingdao Women and Children's Hospital, Qingdao University, No. 6 Tongfu Road, Shibei District, Qingdao, Shandong Province 266000, China; Heart Center, Qingdao Women and Children's Hospital, Qingdao University, No. 6 Tongfu Road, Shibei District, Qingdao, Shandong Province 266000, China

**Keywords:** Coarctation of the aorta, Aortic hypoplasia, Ventricular septal defect, Congenital heart disease, Single-stage surgery, Case report

## Abstract

**Background:**

Coarctation of the aorta (CoA) with a non-restrictive ventricular septal defect (VSD) is a severe congenital heart defect. Traditional single-stage repair often requires deep hypothermic circulatory arrest (DHCA), increasing surgical risk in neonates.

**Case summary:**

A 1-day-old male neonate with prenatal diagnosis of CoA and VSD presented with respiratory distress. Imaging confirmed CoA with arch hypoplasia and a large VSD. He underwent a modified single-stage repair via median sternotomy. The innovative approach involved initial aortic arch reconstruction without cardiopulmonary bypass (CPB), followed by VSD closure under mild hypothermic CPB. The chest was closed primarily. Recovery was remarkable: ventilator support 23 h, intensive care unit stay 4 days, and total hospitalization 13 days. Discharge echocardiogram showed excellent cardiac function with no complications.

**Discussion:**

This modified single-stage approach successfully treated CoA with VSD while avoiding DHCA. The technique’s key advantage lies in performing arch reconstruction without CPB first, significantly reducing CPB duration and eliminating neurological risks associated with DHCA. The excellent postoperative recovery demonstrates this strategy’s potential benefits over conventional methods, offering a promising surgical alternative for managing this complex condition in infants.

Learning points

- Off-pump arch reconstruction followed by on-pump ventricular septal defect (VSD) repair modified procedure corrects coarctation of the aorta/VSD while avoiding deep hypothermic arrest and reducing bypass time.
- Median sternotomy without bypass is feasible for aortic arch reconstruction even with hypoplasia, given adequate mobilization and standby bypass readiness.

## Introduction

Coarctation of the aorta (CoA) combined with ventricular septal defect (VSD) is a common complex congenital heart disease in the neonate.^1^ It is prone to cause intractable congestive heart failure early, requiring timely surgical intervention. Early clinical treatment for that malformation tended towards staged surgery. With advancement in surgical techniques, single-stage surgery has become the mainstream trend.^2–4^ We propose a modified procedure: median sternotomy, aortic arch reconstruction without cardiopulmonary bypass (CPB), followed by conventional CPB for VSD repair. Here, we report a case of a neonate with CoA and VSD treated using this modified procedure.

## Summary figure

**Figure:**
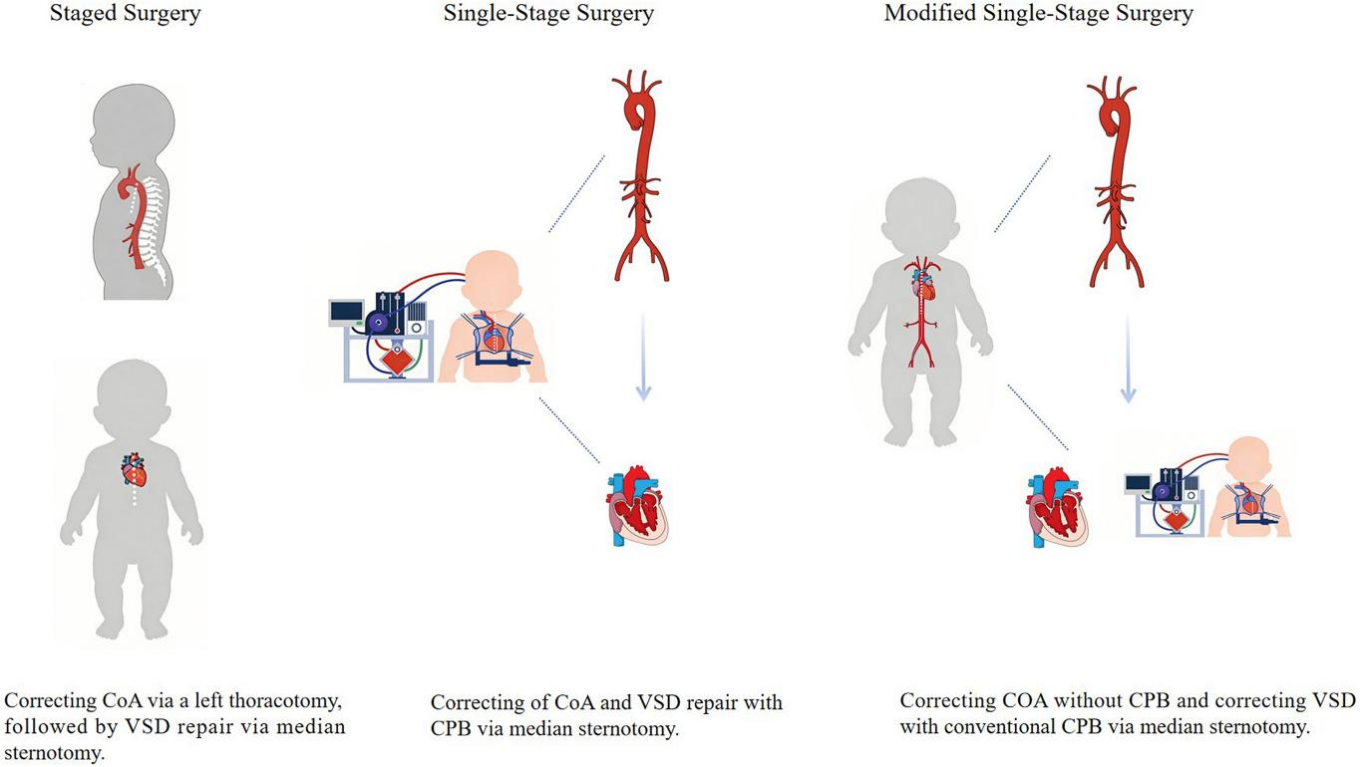


## Case presentation

**Figure 1:**
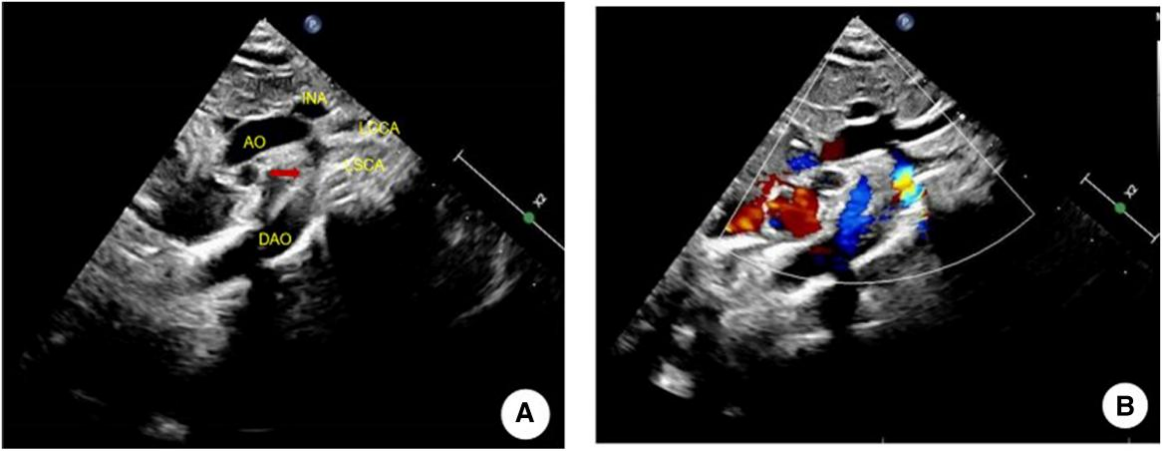
Echocardiogram. (*A*) Red arrow indicates the stenotic site in the descending aorta. (*B*) Fine blood flow through the stenotic site. AO, ascending aorta; INA, innominate artery; LCCA, left common carotid artery; LSCA, left subclavian artery; DAO, descending aorta.

**Figure 2:**
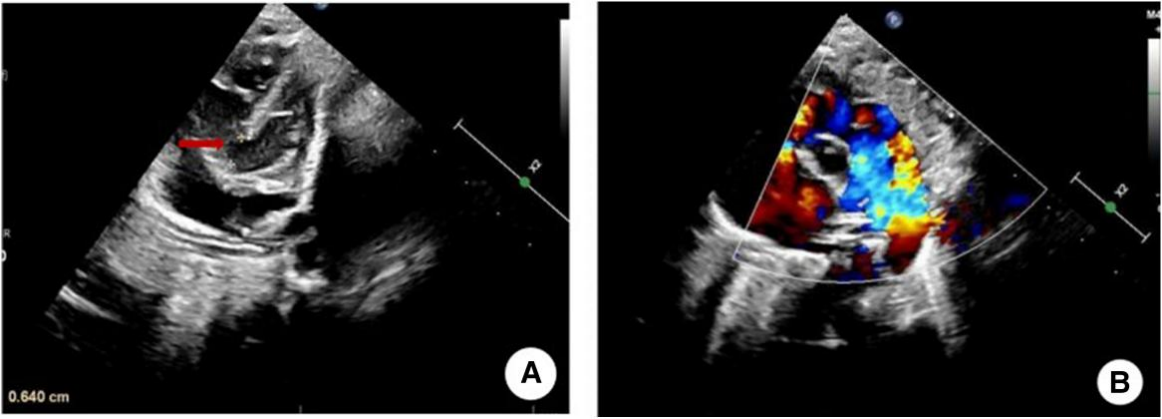
Echocardiogram. (*A*) Red arrow indicates the location of the ventricular septal defect. (*B*) Blood flow showing left-to-right shunt.

A 1-day-old, 3.5 kg male neonate was admitted with prenatal diagnosis of CoA. He developed postnatal respiratory distress. Physical examination revealed tachypnoea, Grade 2/6 systolic murmur, and weakened femoral pulses. Critical haemodynamic findings included significant upper-to-lower-limb blood pressure gradient (upper: 72–78/32–35 mmHg vs. lower: 57–62/26–29 mmHg) and differential cyanosis (pre-ductal SpO_2_ 97% vs. post-ductal 90%). Echocardiography indicated that the transverse aortic arch is hypoplastic and rigid, with significant narrowing at the isthmus (diameter ∼1.6 mm), a 6.7 mm perimembranous VSD with low-velocity left-to-right shunt, and patent ductus arteriosus connecting the descending aorta and pulmonary artery, showing continuous bidirectional shunting and a small left-to-right shunt across the foramen ovale (*Figures 1* and *2*).

**Figure 3:**
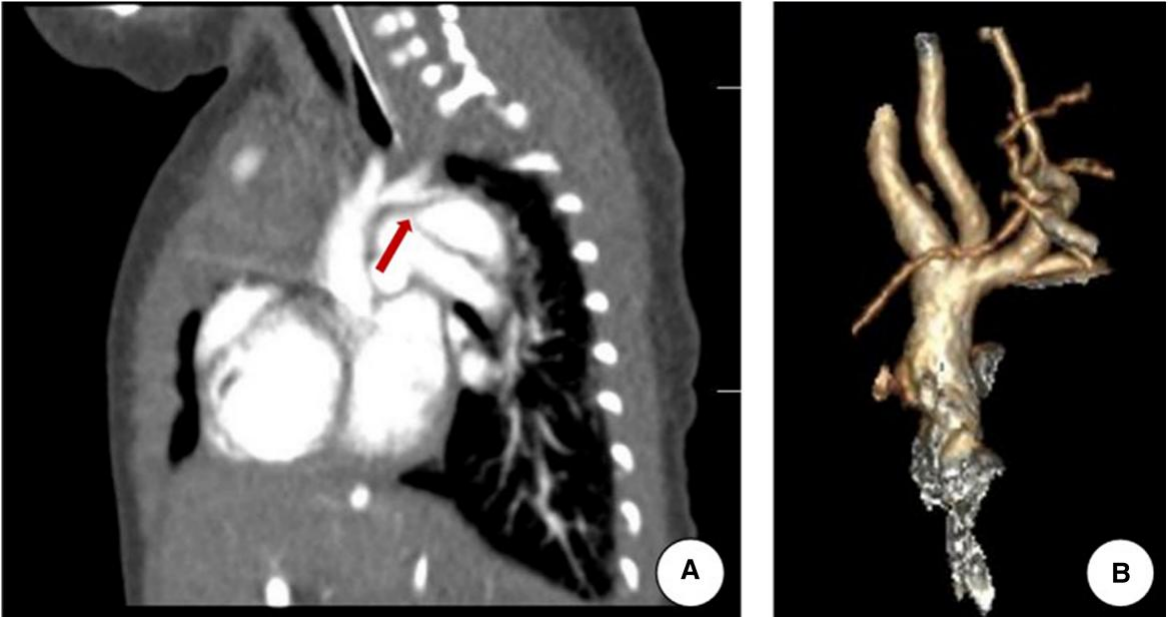
Sagittal and 3D reconstruction images of the computed tomography angiography. (*A*) Sagittal image. (*B*) 3D reconstruction; red arrow indicates arch hypoplasia.

Computed tomography angiography (CTA) demonstrated hypoplastic aortic arch with severe coarctation at the isthmus. The narrowest segment, located distal to the left subclavian artery, measured 2.1 mm in diameter. Perimembranous defect measuring 6.8 mm was confirmed. And the aortic dimensions were notably diminished distal to the brachiocephalic and left subclavian arteries (*Figure 3*).

Following admission, the infant was initiated on an alprostadil infusion to maintain ductal patency. Comprehensive evaluation confirmed the diagnosis of CoA with arch hypoplasia, VSD, atrial septal defect, and patent ductus arteriosus, complicated by moderate pulmonary hypertension. Surgical indication was present. The infant underwent median sternotomy and aortic arch reconstruction without CPB, followed by conventional CPB for VSD repair on the 10th day of life. Surgical steps: first, through a median sternotomy, the ascending aorta, aortic arch, and branches were extensively mobilized, with vessel loops placed. Purse-string sutures were placed on the aorta and right atrium. Then, the stenotic segment of the aortic isthmus was resected, and the ends were oversewn. And the ductus arteriosus was ligated and divided using 6/0 Prolene. Partial occlusion clamps were placed on the proximal arch and descending aorta. Ductal tissue was excised back to normal vessel. An opening was made on the lesser curvature of the proximal aortic arch and anastomosed end-to-side to the descending aorta using a continuous 8/0 Prolene suture. After that, the descending aortic clamp was released first, followed by de-airing and release of the proximal arch clamp (total time 18 min) (see Supplementary material online, *Video S1*). Blood pressure was measured at 65/33 mmHg in the upper limb and 63/32 mmHg at the lower limb. Conventional CPB was established, and cardioplegia was administered. The right atrium was opened longitudinally with cardiac arrest, the VSD was exposed through the tricuspid valve, and the surgical patch was used to repair the VSD with continuous 6/0 Prolene suture. After thorough de-airing and closure of the atrial septal defect, the aortic cross-clamp was released, and the heart resumed sinus rhythm. The right atrium was closed with continuous 6/0 Prolene suture. The patient was weaned from CPB after continued parallel circulation. The aortic cross-clamp time was 32 min, The CPB time was 52 min, the intraoperative urine output was 10 mL, and the total surgery duration was 132 min.

The infant’s chest was closed directly. Postoperative ventilator support lasted 23 h, intensive care unit stay was 4 days, and total hospitalization was 13 days. Pre-discharge echocardiography showed good cardiac function, an aortic anastomotic flow velocity of 1.5 m/s, and no left ventricular outflow tract obstruction.

## Discussion

Historically, a staged surgical approach—initial CoA repair via left thoracotomy followed by VSD closure via sternotomy—was standard due to technical limitations in neonatal CPB and perioperative care.^5,6^ The advantage of this approach is that it avoids the use of CPB in the neonatal period. However, the disadvantages of this approach are outstanding: first, staged surgery requires two separate anaesthetics and surgical traumas; additionally, during the interval between staged surgeries, the persistent left-to-right shunt can lead to ongoing progression of pulmonary hypertension, prolonged postoperative recovery, and high mortality. Some surgeons performed pulmonary artery banding simultaneously with CoA repair to prevent pulmonary hypertension, but this did not yield satisfactory results^7^; finally, for patients with arch hypoplasia, due to the extensive nature of the narrowing, left thoracotomy usually fails to achieve satisfactory correction.

With the development of CPB and perfusion techniques, single-stage repair has gradually become the mainstream procedure, involving median sternotomy with DHCA or selective cerebral perfusion (SCP) to correct the CoA and simultaneously repair the VSD.^8^ Data show that single-stage surgery has no significant difference in operative mortality and complications compared to staged surgery.^9,10^ Although this procedure can correct all anatomical defects, it inevitably uses deep hypothermic circulatory arrest (DHCA) or SCP techniques, and single-stage radical surgery also requires longer CPB times and aortic cross-clamp times. Studies have shown that the duration of DHCA is closely related to postoperative brain injury in neonates.^11^ For SCP techniques, under-perfusion or over-perfusion can damage the nervous system,^12^ and there are reports that SCP does not improve neurodevelopmental outcomes after surgery for complex CHD in infants.^13,14^

In the context of choosing between single-stage and staged surgeries, some scholars innovatively proposed a dual-incision single-stage strategy, performing CoA correction via left thoracotomy followed by VSD repair via median sternotomy with CPB under single anaesthetic.^15^ This approach combines the advantages of single-stage surgery while avoiding the risks associated with DHCA or SCP.^4^

Based on the theoretical foundations of the various aforementioned surgical approaches, we propose a new surgical strategy for infants with CoA and non-restrictive VSD. Through median sternotomy to correct CoA without CPB, followed by VSD repair under conventional CPB. The advantages of this modified procedure are similar to the dual-incision single-stage repair while avoiding the trauma of multiple incisions. It has been suggested that it is difficult to address proximal arch hypoplasia via median sternotomy without CPB.^15^ Prior to implementing this modified technique, we had successfully performed median sternotomy aortic arch reconstruction without CPB for two infants with CoA and arch hypoplasia. Both infants had good postoperative outcomes.

Based on this experience, we regard correction of CoA via median sternotomy without CPB as feasible. The median incision allows for more extensive mobilization of the ascending aorta and branches, avoiding excessive traction and tension on the anastomosis. Simultaneously, the median incision allows for pre-placement of aortic and right atrial purse-string sutures, preparing for potential complications and ensuring surgical safety by having CPB readily available. However, during the operation, the left vagus nerve and the recurrent laryngeal nerve should be fully dissected and isolated to avoid their damage. This can be achieved by having the assistant pull these nerves away from the surgical incision position when dealing with the aortic stenosis segment and cutting the ductus arteriosus, thereby protecting these nerves. The modified procedure avoids the injury associated with DHCA or SCP and the risk of multiple surgeries. It significantly reduced CPB time and aortic cross-clamp time, which is beneficial for postoperative recovery.

## Supplementary Material

ytag098_Supplementary_Data

## Data Availability

The data underlying this article will be shared on reasonable request to the corresponding author.

## Lead author biography

Professor Silin Pan works as the chief physician at the Women and Children’s Hospital, Qingdao University. He is a well-known paediatric cardiologist who has committed to research in the fields of congenital heart disease. He pioneered foetal cardiac intervention and arterial duct stenting for complex heart diseases in China, leading his team to complete the largest series of foetal cardiac surgeries in Asia, with the youngest gestational age being 26 weeks.

## Supplementary material

**Consent:** The authors confirm that written consent for submission and publication of this case report including images and associated text has been obtained from the patient in line with the COPE guidelines.
